# Pessary or Progesterone to Prevent Preterm delivery in women with short cervical length: the Quadruple P randomised controlled trial

**DOI:** 10.1186/s12884-017-1454-x

**Published:** 2017-09-04

**Authors:** Maud D. van Zijl, Bouchra Koullali, Christiana A. Naaktgeboren, Ewoud Schuit, Dick J. Bekedam, Etelka Moll, Martijn A. Oudijk, Wilhelmina M. van Baal, Marjon A. de Boer, Henricus Visser, Joris van Drongelen, Flip W. van de Made, Karlijn C. Vollebregt, Moira A. Muller, Mireille N. Bekker, Jozien T. J. Brons, Marieke Sueters, Josje Langenveld, Maureen T. Franssen, Nico W. Schuitemaker, Erik van Beek, Hubertina C. J. Scheepers, Karin de Boer, Eveline M. Tepe, Anjoke J. M. Huisjes, Angelo B. Hooker, Evelyn C. J. Verheijen, Dimitri N. Papatsonis, Ben Willem J. Mol, Brenda M. Kazemier, Eva Pajkrt

**Affiliations:** 1 Department of Obstetrics and Gynaecology, Academic Medical Center (AMC), Meibergdreef 9, 1105 AZ Amsterdam, The Netherlands; 2 Julius Centre for General Practice and Health Sciences, University Medical Centre Utrecht (UMCU), Utrecht, The Netherlands; 3 Department of Obstetrics and Gynaecology, Onze Lieve Vrouwe Gasthuis (OLVG) Oost, Amsterdam, The Netherlands; 4 Department of Obstetrics and Gynaecology, Onze Lieve Vrouwe Gasthuis (OLVG) West, Amsterdam, The Netherlands; 5 Department of Obstetrics and Gynaecology, Flevoziekenhuis, Almere, The Netherlands; 6 Department of Obstetrics and Gynaecology, VU Medical Centre (VUmc), Amsterdam, The Netherlands; 7 Department of Obstetrics and Gynaecology, Tergooi Hospital, Hilversum, The Netherlands; 8 Department of Obstetrics and Gynaecology, Radboud University Medical Center, Nijmegen, The Netherlands; 9 Department of Obstetrics and Gynaecology, Sint Franciscus Gasthuis, Rotterdam, The Netherlands; 10 Department of Obstetrics and Gynaecology, Spaarne Gasthuis, Haarlem, The Netherlands; 11 Department of Obstetrics and Gynaecology, Spaarne Gasthuis, Hoofddorp, The Netherlands; 12 Department of Obstetrics and Gynaecology, University Medical Centre Utrecht (UMCU), Utrecht, The Netherlands; 13 Department of Obstetrics and Gynaecology, Medisch Spectrum Twente, Enschede, The Netherlands; 14 Department of Obstetrics and Gynaecology, Leiden University Medical Centre (LUMC), Leiden, The Netherlands; 15 Department of Obstetrics and Gynaecology, Zuyderland Hospital, Heerlen, The Netherlands; 16 Department of Obstetrics and Gynaecology, University Medical Centre Groningen (UMCG), Groningen, The Netherlands; 17 Department of Obstetrics and Gynaecology, Diakonessenhuis, Utrecht, The Netherlands; 18 Department of Obstetrics and Gynaecology, Antonius Hospital, Nieuwegein, The Netherlands; 19 Department of Obstetrics and Gynaecology, Maastricht University Medical Centre (MUMC), Maastricht, The Netherlands; 20 Department of Obstetrics and Gynaecology, Rijnstate Hospital, Arnhem, The Netherlands; 21 Department of Obstetrics and Gynaecology, Slingeland Hospital, Doetinchem, The Netherlands; 22 Department of Obstetrics and Gynaecology, Gelre Hospital, Apeldoorn, The Netherlands; 23 Department of Obstetrics and Gynaecology, Zaans Medical Centre (ZMC), Zaandam, The Netherlands; 24 Department of Obstetrics and Gynaecology, Maasziekenhuis Pantein, Boxmeer, The Netherlands; 25 Department of Obstetrics and Gynaecology, Amphia Hospital, Breda, The Netherlands; 26 Robinson Research Institute, School of Paediatrics and Reproductive Health, University of Adelaide, Adelaide, Australia

**Keywords:** Preterm birth, Singletons, Multiples, Prevention, Pessary, Progesterone

## Abstract

**Background:**

Preterm birth is in quantity and in severity the most important topic in obstetric care in the developed world. Progestogens and cervical pessaries have been studied as potential preventive treatments with conflicting results. So far, no study has compared both treatments.

**Methods/design:**

The Quadruple P study aims to compare the efficacy of vaginal progesterone and cervical pessary in the prevention of adverse perinatal outcome associated with preterm birth in asymptomatic women with a short cervix, in singleton and multiple pregnancies separately. It is a nationwide open-label multicentre randomized clinical trial (RCT) with a superiority design and will be accompanied by an economic analysis. Pregnant women undergoing the routine anomaly scan will be offered cervical length measurement between 18 and 22 weeks in a singleton and at 16–22 weeks in a multiple pregnancy. Women with a short cervix, defined as less than, or equal to 35 mm in a singleton and less than 38 mm in a multiple pregnancy, will be invited to participate in the study.

Eligible women will be randomly allocated to receive either progesterone or a cervical pessary. Following randomization, the silicone cervical pessary will be placed during vaginal examination or 200 mg progesterone capsules will be daily self-administered vaginally. Both interventions will be continued until 36 weeks gestation or until delivery, whichever comes first.

Primary outcome will be composite adverse perinatal outcome of perinatal mortality and perinatal morbidity including bronchopulmonary dysplasia, intraventricular haemorrhage grade III and IV, periventricular leukomalacia higher than grade I, necrotizing enterocolitis higher than stage I, Retinopathy of prematurity (ROP) or culture proven sepsis. These outcomes will be measured up until 10 weeks after the expected due date. Secondary outcomes will be, among others, time to delivery, preterm birth rate before 28, 32, 34 and 37 weeks, admission to neonatal intensive care unit, maternal morbidity, maternal admission days for threatened preterm labour and costs.

**Discussion:**

This trial will provide evidence on whether vaginal progesterone or a cervical pessary is more effective in decreasing adverse perinatal outcome in both singletons and multiples.

**Trial registration:**

Trial registration number: NTR 4414. Date of registration January 29th 2014.

**Electronic supplementary material:**

The online version of this article (10.1186/s12884-017-1454-x) contains supplementary material, which is available to authorized users.

## Background

Preterm birth (PTB) is defined as delivery before 37 completed weeks of gestation. PTB affects over 12,000 pregnancies per year in the Netherlands [1] and is the most important cause of perinatal mortality and morbidity, and subsequent neurodevelopmental sequelae. Similarly, neonatal morbidity is strongly increased in case of preterm birth. A follow up study among a cohort of Dutch children born prior to 32 weeks showed that the prevalence of a severe handicap among surviving children was 10% [2].

In women with a singleton pregnancy, the spontaneous preterm birth rate in The Netherlands is around 5%. It results in a perinatal mortality rate in 0.8% and a severe disability rate in 0.7%, while 2% of the children suffer from moderate disability [3]. Women with a multiple pregnancy have a much higher risk of preterm delivery. The incidence of preterm delivery in women with a twin pregnancy is almost 50%, with 1.8, 7 and 14% delivering before 28, 32 and 34 weeks of gestation respectively. As a consequence, women with a twin pregnancy have an 8% perinatal mortality, 7% severely disabled children and 20% moderately disabled children [4]. It is known that a short cervix, bacterial vaginosis, bacteriuria and maternal factors such as maternal periodontal disease, low socio-economic status, smoking and both high and low maternal BMI increase risk of preterm birth [5].

Until a decade ago, there were no effective interventions for the prevention of preterm birth in low-risk pregnancies. However, in the last decade two potentially important breakthroughs have been established: progesterone and pessary. In 2014, an updated Cochrane systematic review reported on the effectiveness of progesterone in the prevention of preterm birth (RR 0.62, 95% CI 0.39–0.98) in women with threatened or established preterm labour, the author’s concluded that due to the low number of included trials, there was insufficient evidence to justify progestational agents as a tocolytic agent for women presenting with preterm labour [6]. However a recent meta-analysis of individual patient data indicated that in asymptomatic women with singleton or multiple pregnancies and a short cervix, progesterone generated lower rates of preterm delivery (26% vs. 36%; OR 0.45, 95% CI 0.25–0.80) and lower rates of perinatal mortality, although this difference did not reach statistical significance (15% vs. 17%; OR 0.69, 95% CI 0.38–1.3) [7].

An individual patient data meta-analysis on 13 trials (7536 children) in multiple pregnancies using progestogens (intramuscular 17-hydroxyprogesterone caproate (17Pc) or vaginal progesterone) showed no overall effect of progestogens on adverse perinatal outcome in twin pregnancies (RR 1.1; 95% CI 0.97–1.4 for 17Pc and RR 0.97; 95% CI 0.77–1.2 for vaginal progesterone) [8]. Nevertheless, vaginal progesterone showed a reduction in adverse perinatal outcome among women with a cervical length equal or below 25 mm (RR 0.57; 95% CI 0.47–0.70 and RR 0.56; 95% CI 0.42–0.75, respectively). The recently published OPPTIMUM trial showed no statistically significant impact on the reduction of spontaneous PTB < 34 weeks or fetal death (OR 0.69; 95% CI 0.39–1.20) or composite adverse neonatal outcome (OR 0.54; 95% CI 0.25–1.16) in the asymptomatic short cervix subgroup. However neonatal death occurred less frequent in the progesterone group (OR 0.17; 95% CI 0.06–0.49). Furthermore, no harm of progesterone was detected when cognitive scores at 2 years of age were compared (−0.48; 95% CI -2.77-1.81) [9].

A second potential breakthrough in the prevention of preterm birth is the use of a cervical pessary [10]. The PECEP trial [11] showed that a cervical pessary reduced preterm birth before 34 weeks in women with a singleton pregnancy and short cervical length (<25 mm) (6% versus 27%). The composite neonatal morbidity did not differ between both groups (RR 0.64; 95% CI 0.27–1.5). Two other RCT’s by Hui et al. and Nicolaides et al. couldn’t reproduce these results. Nicolaides et al. randomized 935 women with a singleton pregnancy to either a cervical pessary or expectant management. The authors found no difference in preterm birth rates before 34 weeks (OR 1.12; 95% CI 0.75–1.69) [12]. The mean gestational age at randomisation was higher in this trial (GA 23 + 5) compared to the trial by Goya et al. (GA 22 + 3) and this might have influenced the effect of the pessary. Within the smaller, prematurely ended, Chinese trial (*n* = 108) birth before 34 weeks occurred in 9.4% and 5.5% in the pessary and expectant management group respectively (*p* = 0.46) [13] Due to slow recruitment and new published results of the PECEP-group, the researchers decided to stop recruitment and report the results of their interim analysis.

In twin pregnancies several studies assessed the effect of pessaries. Within the Dutch Consortium for Healthcare Evaluation and Research in Obstetrics and Gynaecology - NVOG Consortium 2.0, the ProTwin trial was finished, which studied the effectiveness of pessary in unselected twin pregnancies in reducing spontaneous preterm birth and adverse neonatal outcomes. Although there was no overall treatment effect of the pessary, the pessary significantly reduced preterm delivery rates before 32 weeks in a subgroup of women with a cervix less than the 25th percentile (38 mm) (11% vs. 25%, RR 0.44; 95%CI 0.20–0.98). More importantly, within this subgroup, adverse perinatal outcome rate was substantially lower in the pessary group as compared to no intervention (7% vs. 30%, RR 0.23 95% CI; 0.09–0.60) [14]. Long-term follow-up of this study is expected to be published shortly. A more recent trial in women with a twin gestation and a short cervix by Goya et al. revealed a significant reduction of spontaneous preterm birth <34 weeks in the group that received a cervical pessary (RR 0.41 (95% CI 0.22–0.76)). No difference in composite perinatal morbidity and mortality was observed between the groups [15]. The recent trial, by Nicolaides et al., in which 1180 women with a twin pregnancy without a specific cervical length cut-off were allocated to a pessary or expectant management, found no effect of a cervical pessary on preterm birth rates <34 weeks (RR 1.05; 95% CI 0.79–1.4) and adverse neonatal outcome (RR 1.09; 95% CI 0.85–1.4) [16]. The subgroup analysis of 214 women with a cervical length below 25 mm also indicated no differences.

In view of the studies mentioned above, two conclusions can be drawn. First, a short cervix measured at the beginning of the second trimester identifies women at risk for preterm birth [17]. Second, both progestogens and cervical pessary have shown conflicting but promising results for women who are at increased risk for preterm birth due to a short cervix. Both interventions are likely to be introduced in clinical practice since they are non-invasive and potentially cause a cost-reduction.

Until now, no study has directly compared progesterone administration with cervical pessary in a randomized clinical trial (RCT). Therefore, we propose to perform an RCT to compare the efficacy of vaginal progesterone and cervical pessary in the prevention of adverse perinatal outcomes associated with preterm birth in women with a short cervix. The present project is needed to guide the choice between both treatments.

## Methods/Design

### Aims

We will perform a multicenter randomized clinical trial (Pessary or Progesterone to Prevent Preterm delivery in women with short cervical length: the Quadruple P trial) comparing the efficacy of vaginal progesterone capsules and pessary in the reduction of adverse perinatal outcome.

### Participants/eligibility criteria

**Fig. 1:**
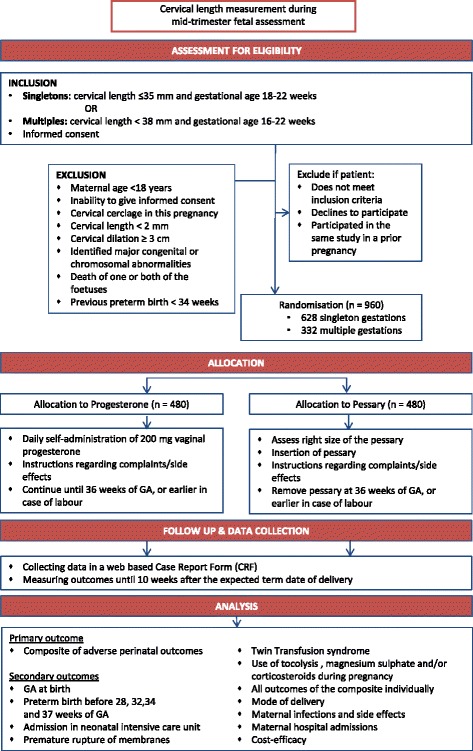
Flow diagram Quadruple P study

Women ≥18 years of age with a singleton or multiple pregnancy (both twin and higher order) undergoing the routine anomaly scan (18–22 weeks for singleton pregnancy and 16–22 weeks for multiple pregnancies) will be offered cervical length measurement. Subsequently we will randomize women with a short cervix, defined as ≤35 mm in singletons and <38 mm in multiples, to a cervical pessary or vaginal progesterone. Both women who receive antenatal care by midwifery practices and women who receive care in the hospital can be included. Exclusion criteria are a previous spontaneous preterm birth before 34 weeks of gestation, a cervical cerclage in this pregnancy, participation in the “Quadruple P study” in a previous pregnancy, identified major congenital abnormalities or death of one or both foetuses, cervical length < 2 mm or cervical dilatation of more than 3 cm. (see Fig. 1).

### Procedures, recruitment and randomization

The study will be performed within the Dutch Consortium for Healthcare Evaluation and Research in Obstetrics and Gynaecology - NVOG Consortium 2.0, a collaboration of approximately 70 obstetric practices (academic and non-academic hospitals) in the Netherlands (www.studies-obsgyn.nl).

All eligible women will be referred to a participating hospital for counseling. Good clinical practice (GCP) trained nurses will counsel patients, ask informed consent, perform randomization and collect data.

Before entry into the study, the investigator or an authorized member of the investigational staff must explain to potential subjects the aims, methods, reasonably anticipated benefits, and potential hazards of the study. Subjects will be informed that their participation is voluntary and that they may withdraw consent to participate at any time. They will be informed that declining participation will not affect their care. Subjects may withdraw at any time or be withdrawn by the investigator if the woman violates the study plan or for administrative and /or safety reasons.

Each subject must give written consent. The subject will be given sufficient time to read the patient information and the informed consent form and gets the opportunity to ask questions. An independent physician will be accessible regarding any questions the subjects may have. The consent form must be signed before any study-related activity can take place. A copy of the informed consent form must be given to the subject. Documents are available through the study website. Patient information is available in Dutch and English and contains information regarding insurance for patients participating in the trial.

Randomization will be centrally controlled using an on-line computerised randomisation service, once patient data have been entered in a web-based database. Centres will be able to access the randomisation service 24 h/day. Subjects will be randomized in a 1:1 ratio to progesterone or a pessary. Figure 1 Randomization will be stratified by centre (to prevent any imbalance between groups in aspects of maternal or neonatal care that may differ between centres) and by singleton/multiple pregnancy. Due to the type of interventions, this study will not be blinded.

Baseline characteristics such as demographic features, obstetric and medical history will be recorded from all randomised women in an electronic case report form (Additional file 1: Appendix 1) in an online database. This database (OpenClinina) is located at a secure server.

### Confidentiality and data security

Initials of subjects and year of birth are recorded in the electronic database. Each participating hospital receives a personal login name and password to access the web-secured database. Linking personal data with randomisation number can only be done in the clinic that performed the randomisation. Full access to the entire database is reserved to some members of the research staff, and must be assigned by the trial bureau and data manager of the NVOG Consortium 2.0.

### Intervention

Participants will be allocated to receive either vaginal progesterone or a cervical pessary (Arabin ®). The cervical pessary will be placed at 18 to 22 weeks in singleton pregnancies and at 16 to 22 weeks in multiple pregnancies, and will stay in situ up to 36 weeks of gestation or until delivery, whatever comes first. It is in the patient’s interest that the pessary is placed by an experienced caregiver to ensure careful placement. In case of complaints, (vaginal) examination is recommended to inspect the cervix and reposition or replace the pessary with another size if necessary. In case of regular contractions, persistent or recurrent vaginal blood loss or premature rupture of the membranes, during treatment with a pessary, the pessary should be removed.

Vaginal progesterone capsules 200 mg will be taken from time of randomisation onwards up to 36 weeks of gestation or delivery, whatever comes first. The capsules will be self-administered vaginally by patients on a daily basis.

The medication will be available at the latest within five working days after randomization. After delivery women will return any unused study medication to the research nurse. The research nurse will count the medication that is left over, and record this in the case record from. Medication that is left over will be destroyed by the pharmacy. The cervical pessary will also be placed within 5 days after randomization and removed during a visit to the hospital at 36 weeks of gestation.

Apart from the research intervention subjects will be treated according to the local protocols in participating clinics and midwifery practices. In case of a threatening preterm delivery other interventions can be performed as usual. Repeating the cervical length measurement is not routinely advised.

### Outcome measures

#### Primary outcome measure

Primary outcome will be composite adverse perinatal outcome including both morbidity and mortality, specifically: severe Respiratory Distress Syndrome (RDS), Bronchopulmonary Dysplasia (BPD), Intraventricular Haemorrhage grade III and IV (IVH), Periventricular Leukomalacia (PVL) higher then grade I, Necrotizing Enterocolitis (NEC) higher then stage I, Retinopathy of Prematurity (ROP) and culture proven sepsis, patent ductus arteriosus (PDA), treated seizures, (intrapartum) stillbirth and death before discharge from the nursery, all measured up until 10 weeks after the expected due date. For the specification of the individual components see Table 1.

**Table 1:** Specification of outcome measurements

*Outcome*	*Defined as*
*Maternal infections*	Two measurements of maternal temperature above 37,8 degrees Celsius at a 1 h interval plus a maternal pulse above 100 beats/min requiring treatment with antibiotics
*Maternal side effects*	Vaginal bleeding, discharge, discomfort and dyspareunia
*BPD (*severe chronic lung disease)	Babies born before 32 weeks: need for >30% oxygen, with or without positive pressure ventilation or continuous positive pressure at 36 weeks postmenstrual age, or discharge (whichever comes first). Babies born after 32 weeks: need for >30% oxygen with or without positive pressure ventilation or continuous positive pressure at 56 days postnatal age, or discharge (whichever comes first).
*IVH > grade II*	Haemorrhage in the germinal matrix, ventricles, or cerebral parenchyma; observed on ultrasound examination or MRI
*PVL > grade I*	periventricular lucency in the white matter
*NEC > stage I*	The presence of the characteristic clinical features of abdominal distention, with or without rectal bleeding, and abdominal radiographic finding associated with pneumatosis intestinalis
*Early sepsis*	If prior to or at 72 h of life the infant had an infection marked by positive blood, CSF, or urine (catheterized or suprapubic) cultures with or without suspicious clinical findings of infection on physical examination.
*Late sepsis*	If after 72 h of life the infant had an infection marked by positive blood, CSF, or urine (catheterized or suprapubic) cultures with or without suspicious clinical findings of infection on physical examination OR If there is clinical evidence of cardiovascular collapse or an unequivocal X-ray confirming infection and often cardiovascular decomposition
*Neonatal meningitis*	Suspected or proven (caused by any pathogen)

#### Secondary outcome measures

Secondary outcomes will be time between randomisation and delivery, preterm birth rate before 28, 32, 34 and 37 weeks (spontaneous, iatrogenic and total), birth weight, (days of) admission in neonatal intensive care unit, premature rupture of the membranes (PPROM), tocolysis (duration), use of corticosteroids, use of magnesium sulphate, mode of delivery, Twin Transfusion Syndrome (TTS), maternal morbidity, maternal admission days for preterm labour. In addition, all components of the primary outcome will also be assessed separately as a secondary outcome measure. A cost-effectiveness analysis will be performed and reported separately from the primary trial results.

Information on the delivery, maternal and fetal condition and admission to the hospital of both mother and child (ren) will be recorded in the electronical case record form. (Additional file 1: Appendix 1).

### Follow-up of women and infants

The outcome measures will be measured until 10 weeks after expected date of delivery.

The only reason for not obtaining complete information is that the patient was lost to follow up or that she withdrew consent to access her medical chart after delivery. If a woman refuses to complete her follow-up visits with the research nurse (RN), the RN will confirm permission to consult her hospital chart in order to be able to complete information on the primary outcome of the study. In this case, her data will be considered in the final analysis.

At present, no long-term follow-up is planned. However, if a sufficient budget is awarded the possibilities to perform a long-term follow-up will be assessed. This will be added to the trial documents using an amendment. Permission to approach patients for follow-up research will be asked via the informed consent.

## Statistical issues

### Sample size

The study was designed as a superiority trial comparing the efficacy of vaginal progesterone and pessary in the prevention of adverse perinatal outcome.

In singleton pregnancies we expect a reduction of adverse perinatal outcome from 5% in the vaginal progesterone group to 1% in the pessary group. The proportion of adverse perinatal outcome of 5% in the vaginal progesterone is based on the Triple P study [18] which was performed in a comparable population. The frequency of adverse perinatal outcome of 1% in the pessary group is based on the PECEP trial where a 3% poor perinatal outcome was found [11]. However the PECEP trial included women with a cervix below 25 mm only. Since we include women with a cervix below or equal 35 mm, we expect a lower frequency of adverse perinatal outcome in our study population. Using a two-sided test with a type I error of 5% and type II error of 20% and a loss to follow-up of 10%, we calculated that we would need a sample size of 628 women (314 per group).

For multiple pregnancies we expected frequencies of adverse perinatal outcome of 24% per pregnancy in the vaginal progesterone group and 12% in the pessary group. In the IPD meta-analysis of Schuit et al. [8] adverse perinatal outcome was 25% in the progesterone-group, in a population with a shorter cervix (< 25 mm). In the ProTwin trial adverse perinatal outcome was 12% in a comparable population with a similar cervical length [14]. We expect a 50% reduction of adverse perinatal outcome in the pessary group. Since multiple gestations are followed up in the hospital, less patients being lost to follow up were expected. Using a two-sided test with a type I error of 5% and type II error of 20% and a loss to follow-up of 5%, a sample size of 332 women (166 per group) would be needed to be able to detect a significant difference.

### Data analysis

All analyses will be performed for singleton and multiple pregnancies separately and will be according to the intention to treat principle. The primary outcome, that is adverse perinatal outcome, will be assessed by calculating and comparing rates in the two groups, using a random intercept fixed effects binomial model with a log link function, resulting in a relative risk and 95% confidence intervals. The random intercept is used to account for the stratified randomization by centre. In multiple pregnancies the clustering of children within one mother will be taken into account in the analysis using generalised estimating equations instead of a log-binomial model [19]. Numbers needed to treat will be calculated when appropriate.

To evaluate the potential of each of the strategies, we will also perform a per protocol analysis, taking into account only those cases that were treated according to protocol.

#### Secondary study parameter(s)

Time to delivery will be evaluated by Kaplan-Meier estimates, with account for different durations of gestation at entry, and will be tested with the log rank test. Again, stratified randomisation will be taken into account by incorporating centre as a stratification variable. The other secondary outcome measures will be approached similarly to the primary outcome measure. Differences in continuous outcomes between both strategies will also be assessed using a random intercept fixed effects linear regression model.

We plan a pre-specified subgroup analysis based on cervical length (below 25 mm vs. equal to and above 25 mm, and below the 25th percentile vs. between the 25th and 50th percentile vs. above the 50th percentile), parity (nulliparous vs. multiparous), previous preterm birth between 34 and 37 weeks (yes vs. no), chorionicity (monochorionic vs. dichorionic; multiple pregnancies only), and number of multiples (twins vs. higher order). Subgroup analysis will be performed by including an interaction term between the subgrouping variables and the treatment allocation (pessary vs. vaginal progesterone). When the interaction will be found statistically significant (*p* < 0.05) we will estimate the treatment effect within the different strata of the subgroup.

### Interim analysis and safety

All AEs will be recorded, either after spontaneous report by the participant or after observation by the investigator and his staff. AEs will be followed until they have abated, or until a stable situation has been reached. Depending on the event, follow up may require additional tests or medical procedures as indicated, and/or referral to the general physician or a medical specialist.

SAEs need to be reported till end of study, as defined in the protocol.

SAEs will be reported through the web portal *ToetsingOnline* to the accredited Medical Ethical Committee (MEC) that approved the protocol.

An interim analysis for effectiveness will not be performed. In case of a strong positive effect of one of the investigated interventions, the trial will be continued. Negative effects will be detected by the data safety monitoring committee based on the SAE’s in both treatment arms.

An interim safety review is planned after all outcomes are available for 320 patients. Results of these safety reviews will be reported separately for singleton and multiple pregnancies. On the basis of these results, the Data Safety Monitoring Committee (DSMC) can decide to perform additional safety reviews. Serious events that may cause concern about the safety of the study (such as maternal mortality); will be reported to the DSMC immediately if they occur.

The DSMC can advise to stop the trial at any moment when the safety of the patients is considered to be in danger based on the SAE reporting.

## Discussion

Prevention of PTB remains one of the main goals in obstetric care. Over the past years both mechanical interventions such as a cervical pessary and pharmacological interventions such as progesterone were evaluated.

Vaginal progesterone as well as a pessary have been shown to be potentially effective measures for the prevention of preterm birth. Both interventions are relatively simple and can be combined with regular care. However, up to now, no study has directly compared treatment with progesterone and cervical pessary.

At this moment, other international study groups have also set up trials to assess both progesterone and cervical pessaries as a preventive strategy for preterm birth. Pooling data of these trials can hopefully help to conclude which intervention is most efficacious in preterm birth prevention. Ongoing (prospective) individual participant meta-analysis on progesterone and pessaries (PROMPT) in singletons and multiples will hopefully give new answers on how to prevent spontaneous preterm deliveries. Protocols of the different ongoing pessary trials have committed to use the same outcome variables [20].

## Additional files

Additional file 1: Figure 1. Flow diagram Quadruple P study. (PDF 402 kb)

Additional file 2: Table 1. Specification of outcome measures. (PDF 129 kb)

## Abbreviations

17 PC: Intramuscular 17-hydroxyprogesterone caproate
AMPHIA: 17-Alpha hydroxyprogesterone in Multiple pregnancies to Prevent Handicapped InfAnts trial
BPD: Bronchopulmonary Dysplasia
CRF: Case Report Form
DSMC: Data Safety Monitoring Committee
IVH: Intraventricular Haemorrhage grade III and IV
MEC: Medical Ethical Committee
NEC: Necrotizing Enterocolitis
PECEP: Pesario Cervical para Evitar Prematuridad trial
PPROM: Premature rupture of the membranes
PROMPT: Prospective Meta-analysis for Pessary Trials
PROTWIN: Pessaries in multiple pregnancy as a prevention of preterm birth study
PTB: Preterm birth
PVL: Periventricular Leucomalacia
Quadruple P: Pessary or Progesterone to Prevent Preterm delivery in women with short cervical length trial
RDS: Respiratory Distress Syndrome
RN: Research nurse
ROP: Retinopathy of prematurity
SAE: Serious adverse events
Triple P: Preventing Preterm birth with Progesterone study
