# Supplementary material for: Pessary or Progesterone to Prevent Preterm delivery in women with short cervical length: the Quadruple P randomised controlled trial
Source: BMC Pregnancy Childbirth. 2017 Sep 4;17:284. doi: 10.1186/s12884-017-1454-x (PMC5584011; doi:10.1186/s12884-017-1454-x)
Supplement: Supplementary file 1 — Flow diagram Quadruple P study. (PDF 402 kb) [file 12884_2017_1454_MOESM1_ESM.pdf]

Quadruple P

Confidential
Case report form

Comparing the effectiveness of vaginal progesterone and cervical pessary in the prevention of preterm birth in women with singleton and twin pregnancies with a short cervix: a multicentre randomised trial.

Case number: ☐☐-☐☐☐☐
Patient initials: ☐☐

This is a CRF handout for the purpose of support for the Quadruple P website.

CRF data entry, information and randomisation:
http://www.studies-obsgyn.nl/

Coordinating researcher: M. van Zijl
Principal investigator: E. Pajkrt, gynaecologist

Table of contents

1. General…………………………………………………………………………………3
1.1. General information…………………………………………………………......... 3
1.2. Inclusion criteria…………………………………………………………….........4
1.3. Exclusion criteria………………………………………………………………… 4

2. Randomisation………………………………………………………………………5

3. Demographics…………………………………………………………………… 6

4. Medical history……………………………………………………………………….7

5. Obstetric history……………………………………………………………………...8

6. Current pregnancy…………………………………………………………………...9
6.1. Start of pregnancy……………………………………………………………….....9
6.2. Laboratory results at study entry……………………………………………….. 9
6.3. Fetal data at study entry…………………………………………………………9
6.4. Transvaginal ultrasound…………...………………………………………………..9

7. Intervention………………………………………………………………………..10
7.1. Pessary or progesterone..………………………………………………………... 10

8. Admissions and complications……………………………………………………..13
8.1. Pregnancy complications after randomisation……………………………………..13
8.2 Admissions afterandomization………………………………………………………14

9. Delivery…………………………………………………………………………………15
9.1. Onset of labour/induction…………………………………………………………15
9.2Delivery……………………………………………………………………………

10. Neonatal data post-partum…………………………………………………………..18

11. Post partum admission………………………………………………………… 20
11.1. Maternal admission……………………………………………………...................20
11.2. Neonatal admission……………………………………………………………… 21

12. Neonatal diagnosis……………………....……………………………………………23

13. Serious Adverse Event
………………………………………………………………………………
21
27

TO BE FILLED OUT AT STUDY ENTRY:

1. General

1.1. General information

Clinic: ☐☐

02 = Academisch Medisch Centrum, Amsterdam
34 = Amphia Ziekenhuis, Breda
05 = Atrium Medisch Centrum, Heerlen
57 = Bethesda Ziekenhuis, Hoogeveen
58 = BovenIJ Ziekenhuis, Amsterdam
21 = Bronovo Ziekenhuis, Den Haag
50 = Canisius Wilhelmina Ziekenhuis, Nijmegen
16 = Catharina Ziekenhuis, Eindhoven
14 = Deventer Ziekenhuis, Deventer
32 = Diakonessenhuis, Utrecht
24 = Elkerliek ziekenhuis, Helmond
47 = Erasmus Medisch Centrum, Rotterdam
51 = Flevoziekenhuis, Almere
15 = Gelre Ziekenhuis, Apeldoorn
35 = Ikazia Ziekenhuis, Rotterdam
45 = Isala Klinieken, Zwolle
20 = Jeroen Bosch Ziekenhuis, ‘s Hertogenbosch
70 = Kennemer Gasthuis, Haarlem
01 = Leids Universitair Medisch Centrum, Leiden
04 = Maastricht Universitair Medisch Centrum, Maastricht
08 = Martini Ziekenhuis, Groningen
07 = Máxima Medisch Centrum, Veldhoven
33 = Meander Medisch Centrum, Amersfoort
29 = Medisch Centrum Alkmaar
27 = Medisch Centrum Leeuwarden
36 = Medisch Spectrum Twente, Enschede
78 = Nij Smellinghe ziekenhuis, Drachten
09 = Onze Lieve Vrouwe Gasthuis, Amsterdam
48 = Orbis Medisch Centrum, Sittard-Geleen
43 = Radboud Universitair Medisch Centrum, Nijmegen
81 = Rijnland - Alphen ad Rijn / Leiderdorp
39 = Rijnstate, Arnhem
85 = Ruwaard van Putten ziekenhuis - Spijkenisse
37 = Scheper Ziekenhuis, Emmen
11 = Sint Antonius Ziekenhuis, Utrecht
13 = Sint Elisabeth Ziekenhuis, Tilburg
40 = Sint Lucas Andreas Ziekenhuis, Amsterdam
30 = Spaarne Ziekenhuis, Hoofddorp
65 = Tergooiziekenhuis, Blaricum
06 = TweeSteden ziekenhuis, Tilburg
03 = Universitair Medisch Centrum Groningen
12 = Universitair Medisch Centrum Utrecht
26 = VieCuri Medisch Centrum voor Noord-Limburg, Venlo
28 = Zaans Medisch Centrum – Zaandam
94 = Ziekenhuisgroep Twente, Almelo

Case number: ☐☐-☐☐☐☐

Composition of pregnancy:
O Singleton
O Twin

Case number: ☐☐ - ☐☐☐☐ (clinic - case)
Date of birth: ☐☐-☐☐-☐☐ (dd-mm-yy)
Estimated due date ☐☐-☐☐-☐☐ (dd-mm-yy)

1.2. Inclusion criteria

Inclusion criteria*		No	Yes
1.	Singleton pregnancy and cervical length of 35 mm or less (≤ 35 mm) at 18+0 to 21+6 weeks<br><br>OR<br><br>Twin pregnancy and cervical length of 38 mm or less (≤ 38 mm) at 16+0 to 21+6 weeks	O	O

1.3. Exclusion criteria

Maternal/Fetal Exclusion criteria		No	Yes
		O	O
	Cervical cerclage in this pregnancy	O	O
	Maternal age less than 18 years	O	O
	Cervical dilatation ≥ 3 cm	O	O
	Identified major congenital abnormalities #	O	O
	Death of one or both of the foetuses	O	O
	History of preterm singleton birth before 34 weeks of gestation	O	O
	Participation Quadruple P study in previous pregnancy	O	O

# Major fetal abnormalities are defined as those that are lethal or require intensive prenatal care or postnatal surgery.

2. Randomisation

Randomisation result:

O Pessary
O Progesteron

O No randomization (observational cohort)
|
If consent for observational cohort signed:

Estimated due date □□-□□-□□ (dd-mm-yy)

Child

Live birth
O Yes
O Termination of pregnancy
O No, deceased before labor
O No, deceased during labor

Date of birth or date of diagnosis of death	□□-□□-□□ (dd-mm-yy)

Gender: O Boy O Girl

Birth weight □□□□ (kg)

Did patient receive progesterone? O yes O no |
|
If yes start date:
stop date:

Did patient receive a pessary? O yes O no |
|
If yes start date:
stop date:

Has there been complication related to the pessary or progesterone?
If yes please specify:………………………..

TO BE FILLED OUT AFTER RANDOMISATION:

3. Demographics

Ethnicity:

O Caucasian
O Indian/ Pakistani/ Bangladesi
O Afro-Caribbean (Antillen, Suriname –creool)
O Hindu, Caribbean (Suriname – hindu)
O African (sub-Sahara)
O Middle-East + North Africa (Turkish and Moroccan)
O Asian
O Other
O Unknown

Highest finished education:

O Primary school	(basisschool)
O Secondary school	(havo, VWO, gymnasium)
O Lower professional school	(VMBO)
O Medium professional school	(MBO)
O Higher professional school	(HBO – post HBO)
O University	(WO)
O Unknown	

4. Medical history

Diseases:

Diabetes Mellitus	O no	O yes
Hypertension	O no	O yes
Depression	O no	O yes
Renal insufficiency	O no	O yes
Inflammatory bowel disease	O no	O yes
Thrombophilia	O no	O yes
Thyroid disease	O no	O yes

Risk factors for preterm delivery:

Conisation/ LETTZ	O no	O yes	O unknown
Uterus anomaly	O no	O yes	O unknown
Known uterine surgery	O no	O yes	O unknown

Smoking O no O yes O quitted in first trimester
O unknown | |
If yes or quitted,
Amount: O ≤ 10 sig/day
O > 10 sig/day
O unknown

Treated urinary tract infections in past year
O 0 O 1 O>1 O unknown

Case number: ☐☐-☐☐☐☐

5. Obstetric history

Gravidity ☐☐ ; Parity ☐☐; Miscarriage/abortion/ectopic pregnancy* ☐☐

* < 16 weeks

Was there any curettage?
O no O yes O unknown

Missing value (unknown) = -1
If the obstetric history contains a preterm delivery < 34 weeks, then the patient cannot be randomized.

Route:
0 = spontaneously
1 = vacuum
2 = forcipal extraction
3 = caesarian (secondary)
-1 = unknown

Birth weight 50-6000 gram

Start:
0 = spontaneously
1 = induction
2 = caesarian (primary)
3 = curettage
-1 = unknown

GA: weeks + days

Date of birth: XX/'XX

	Date of birth Month/Year	GA	Start	Route	Birth weight	If twin: Birth Weight Baby 2
1.	☐☐/☐☐	☐☐+☐	☐	☐	☐☐☐☐	☐☐☐☐
2.	☐☐/☐☐	☐☐+☐	☐	☐	☐☐☐☐	☐☐☐☐
3.	☐☐/☐☐	☐☐+☐	☐	☐	☐☐☐☐	☐☐☐☐
4.	☐☐/☐☐	☐☐+☐	☐	☐	☐☐☐☐	☐☐☐☐
5.	☐☐/☐☐	☐☐+☐	☐	☐	☐☐☐☐	☐☐☐☐
6.	☐☐/☐☐	☐☐+☐	☐	☐	☐☐☐☐	☐☐☐☐
7.	☐☐/☐☐	☐☐+☐	☐	☐	☐☐☐☐	☐☐☐☐
8.	☐☐/☐☐	☐☐+☐	☐	☐	☐☐☐☐	☐☐☐☐
9.	☐☐/☐☐	☐☐+☐	☐	☐	☐☐☐☐	☐☐☐☐
10.	☐☐/☐☐	☐☐+☐	☐	☐	☐☐☐☐	☐☐☐☐
11.	☐☐/☐☐	☐☐+☐	☐	☐	☐☐☐☐	☐☐☐☐
12.	☐☐/☐☐	☐☐+☐	☐	☐	☐☐☐☐	☐☐☐☐
13.	☐☐/☐☐	☐☐+☐	☐	☐	☐☐☐☐	☐☐☐☐
14.	☐☐/☐☐	☐☐+☐	☐	☐	☐☐☐☐	☐☐☐☐

6. Current pregnancy

6.1. *Start of pregnancy*

Mode of conception:

- O Spontaneous
- O IUI
- O Ovulation induction
- O IVF
- O ICSI

Height: □□□ cm (140-210)
Weight before pregnancy □□□ kg (40-250)

6.2. *Laboratory results at study entry*

GBS carrier

GBS carrier (known prior to pregnancy) O no O yes O unknown

6.3. *Fetal data at study entry*

Composition of pregnancy:

- O Singleton
- O Twin, diamniotic, dichrionic
- O Twin, diamniotic, monochorionic
- O Twin, monoamniotic, monochrionic
- O Twin, chorionicity unknown
- O Triplet, triamniotic, trichrionic
- O Triplet, dichorionic diamniotic
- O Triplet,dichorionic triamniotic
- O Triplet, monochorionic, triamniotic
- O Triplet, monochorionic, diamniotic
- O Triplet, monochorionic, monoamniotic
- O Triplet, chorionicity unknown

6.4. *Transvaginal Ultrasound*

<u>Transvaginal ultrasound at admission</u>:

Date of cervical length measurement	□□-□□-□□ (dd-mm-yy)
Cervical length at randomisation	□□ mm (≥5 to 38)
Funneling	O no O yes O unknown
Sludge	O no O yes O unknown

7. Intervention

7.1. *Pessary or progesterone*

Randomisation result O pessary O progesterone

When randomized to pessary

Did patient receive a pessary: O no O yes

|
O cervical length too small
O pessary size not in stock
O woman refused pessary placement
O delivered before placement
O patient preferred progesterone
O doctor preferred progesterone
O unknown
O

Date of placing pessary ☐☐-☐☐-☐☐ (dd-mm-yy)

Size pessary

O Small	65x25x32
O Medium (standard)	70x25x32
O Large	70x25x35
O unknown/other	

When was the pessary removed?

Date: ☐☐ - ☐☐ - ☐☐ (dd-mm-yy)

Why was the pessary removed?

☐ GA > 36 weeks
☐ vaginal pain
☐ excessive discharge
☐ (P)PROM
☐ vaginal blood loss
☐ contractions/labor
☐ Pessary fell out
☐ Require delivery for other reasons
☐ patient changed to progesterone

If the pessary was removed, was it replaced?

O no O yes O unknown
If yes,
Date: ☐☐ - ☐☐ - ☐☐ (dd-mm-yy)

Case number: □□-□□□□

Was the second pessary removed?

O no O yes O unknown

|

- □ GA > 36 weeks
- □ vaginal pain
- □ excessive discharge
- □ (P)PROM
- □ vaginal blood loss
- □ contractions/labor
- □ Pessary fell out
- □ Require delivery for other reasons
- □ patient changed to progesterone

Final date of removal pessary:

Date: □□ - □□ - □□ (dd-mm-yy)

When randomized to progesterone

Did patient start with progesterone: O no O yes

|

- O woman refused use of progesterone
- O delivered before starting
- O patient preferred a pessary
- O doctor preferred a pessary
- O unknown

Date of starting progesterone therapy □□-□□-□□ (dd-mm-yy)

Type of progesterone:

- O Vaginal suppository
- O Gel
- O Injection

Dose of progesterone:

- O 80 mg
- O 100 mg
- O 200 mg
- O 250 mg
- O other/unknown

When did the patient stop with the progesterone therapy?

Date: ☐☐ - ☐☐ - ☐☐ (dd-mm-yy) #

# actual stopdate O unknown Why did the patient stop with progesterone therapy?

- ☐ GA > 36 weeks
- ☐ vaginal pain
- ☐ discharge
- ☐ PPROM
- ☐ vaginal blood loss
- ☐ contractions/labor
- ☐ Require delivery for other reasons
- ☐ patient changed to a pessary
- ☐ other

If the therapy was stopped, was it restarted?

O no O yes O unknown

If yes,

Date: ☐☐ - ☐☐ - ☐☐ (dd-mm-yy)

Did the patient stop again with the progesterone therapy?

O no O yes O unknown

|

- ☐ GA > 36 weeks
- ☐ vaginal pain
- ☐ excessive discharge
- ☐ (P)PROM
- ☐ vaginal blood loss
- ☐ contractions/labor
- ☐ Require delivery for other reasons
- ☐ patient changed to a pessary
- ☐ other

How many capsules did the patient receive?

O 0 O 70 O 140 O unknown

How many capsules were send back by the patient?

O none ☐☐☐☐ (amount)

Final date of stop progesterone therapy.

Date: ☐☐ - ☐☐ - ☐☐ (dd-mm-yy)

8.1 Pregnancy complications after randomization

Treated genital tract infection	O no	O yes	O unknown
Treated urinary tract infection	O no	O yes	O unknown
Ruptured membranes <36 weeks	O no	O yes<br>| If yes, Date:<br>□□ - □□ - □□ (dd-mm-yy)	O unknown
Excessive vaginal discharge	O no	O yes	O unknown
Vaginal blood loss<br>*not related to placing pessary*	O no	O yes	O unknown
Pelvic pain<br>*not related to placing pessary*	O no	O yes	O unknown

Chorioamnionitis
Requires 2 of the following: fever, fundal tenderness, maternal tachycardia, fetal tachycardia, foul-smelling amniotic fluid

O no O yes O unknown

Twin Twin Transfusion syndrome

O no O yes O unknown

Case number: □□-□□□□

8.2 Admissions after randomization

1= Threatened preterm birth
2= PE
3= IUGR
4= Complication of placing pessary
5= Decreased fetal movement
6= Labor/induction
7= Vaginal bleeding
8= Other
10=unknown

Fill in the details for number of admissions:

1= Ward
2= Medium care
3= Intensive care
4= home monitoring

Admission number	Date of admission to hospital (dd-mm-yy)	Date of discharge from hospital (dd-mm-yy)	Type of admission	Primary indication for admission
1	□□ - □□ - □□	□□ - □□ - □□		
2	□□ - □□ - □□	□□ - □□ - □□		
3	□□ - □□ - □□	□□ - □□ - □□		
4	□□ - □□ - □□	□□ - □□ - □□		
5	□□ - □□ - □□	□□ - □□ - □□		
6	□□ - □□ - □□	□□ - □□ - □□		
7	□□ - □□ - □□	□□ - □□ - □□		
8	□□ - □□ - □□	□□ - □□ - □□		

9. Delivery

9.1Onset of labor/induction

Start date labor onset: □□-□□-□□ (dd-mm-yy)

Onset of labor:

O Spontaneous

O Induction →

If induction, reason:
- □ maternal indication
- □ fetal indication
- □ ruptured membranes
- □ elective (also term >41 weeks

O Primary C-Section

9.2 Delivery

Birth child:	Date	□□-□□-□□ (dd-mm-yy)
	Time	□□:□□ (hh-mm)
Child 2:	Date	□□-□□-□□ (dd-mm-yy)
	Time	□□:□□ (hh-mm)
Child 3:	Date	□□-□□-□□ (dd-mm-yy)
	Time	□□:□□ (hh-mm)

Route of delivery
- O Spontaneously
- O Vacuum extraction
- O Forcipal extraction
- O Caesarean section

Indication vacuum/ forceps?
- □ Fetal distress
- □ Maternal indication
- □ Failure to progress in second stage

Indication Caesarean section?
- □ Failure to progress, first stage
- □ Failure to progress, second stage
- □ Failed instrumental delivery
- □ Fetal distress
- □ Elective reason
- □ Maternal indication

Presentation at birth
- O Cephalic
- O Breech

O Transverse

Did mother require antibiotics in labor for treatment of suspected infection (not prophylaxis)? O no O yes O unknown

Dis mother receive tocolysis?
` O no O yes O unknown

Did mother received corticosteroids?
O no O yes O unknown

Did mother receive magnesiumsulfate?
O no O yes O unknown

"Did the patient ever receive a cerclage? O no O yes date

In case of cerclage, date removal stitches ☐☐-☐☐-☐☐ (dd-mm-yy)

Treated postpartum endometritis or pelvic infection? O no O yes

10. Neonatal data post-partum

Child 1

Live birth
O Yes
O No, termination of pregnancy
O No, deceased before labor
O No, deceased during labor

Date of diagnosis of death ☐☐-☐☐-☐☐ (dd-mm-yy)

Gender: O Boy O Girl

Apgar-scores:

1 min ☐☐ (0–10)

5 min ☐☐ (0–10)

Weight at birth ☐☐☐☐ gr. (0 – 6000)

Umbilical cord measurements: (pH: 6.00-7.70; BE: -30.0-+10.0; unknown: -1)

Art. pH ☐.☐☐ Art. BE ☐☐.☐

Ven. pH ☐.☐☐ Ven. BE ☐☐.☐

Child 2:

Live birth
O Yes
O Termination of pregnancy
O No, deceased before labor
O No, deceased during labor

Date of diagnosis of death ☐☐-☐☐-☐☐ (dd-mm-yy)

Gender: O Boy O Girl

Apgar-scores:

1 min ☐☐ (0–10)

5 min ☐☐ (0–10)

Weight at birth ☐☐☐☐ gr. (0 – 6000)

Umbilical cord measurements: (pH: 6.00-7.70; BE: -30.0-+10.0; unknown: -1)

Art. pH	☐.☐☐	Art. BE	☐☐.☐
Ven. pH	☐.☐☐	Ven. BE	☐☐.☐

Child 3:

Live birth
O Yes
O Termination of pregnancy
O No, deceased before labor
O No, deceased during labor

Date of diagnosis of death ☐☐-☐☐-☐☐ (dd-mm-yy)

Gender: O Boy O Girl

Apgar-scores:

1 min ☐☐ (0–10)

5 min ☐☐ (0–10)

Weight at birth ☐☐☐☐ gr. (0 – 6000)

Umbilical cord measurements: (pH: 6.00-7.70; BE: -30.0-+10.0; unknown: -1)

Art. pH	☐.☐☐	Art. BE	☐☐.☐
Ven. pH	☐.☐☐	Ven. BE	☐☐.☐

Fetal anomaly O no O yes

Specify………

11. Postpartum Admission

Were mother or child admitted directly postpartum?

O No, there was no admission – END OF CRF
O Yes, maternal admission only
O Yes, neonatal admission only
O Yes, maternal and neonatal admission
If yes: O maternal indication O neonatal indication O mother and child

11.1 Maternal Admission

Admission #1

Type of admission O Ward
O Medium care
O Intensive care

Discharge to O Home Complete "Date of final discharge to home"
O Other ward ------▶Transfer date ☐☐-☐☐-☐☐ (dd-mm-yy)
O Other hospital

Admission #2

Type of admission O Ward
O Medium care
O Intensive care

Discharge to O Home Complete "Date of final discharge to home"
O Other ward ------▶Transfer date ☐☐-☐☐-☐☐ (dd-mm-yy)
O Other hospital

Admission #3

Type of admission O Ward
O Medium care
O Intensive care

Discharge to O Home Complete "Date of final discharge to home"
O Other ward ------▶Transfer date ☐☐-☐☐-☐☐ (dd-mm-yy)
O Other hospital

Maternal death O No O Yes

date □□-□□-□□ (dd-mm-yy)

Date of final discharge to home □□-□□-□□ (dd-mm-yy)

11.2 Neonatal Admission

Admission #1

Type of admission
- O Ward
- O Medium care
- O Neonatal intensive care unit

Discharge to
- O Home → Complete "Date of final discharge to home"
- O Other ward → Transfer date □□-□□-□□ (dd-mm-yy)
- O Other hospital

Admission #2

Type of admission
- O Ward
- O Medium care
- O Neonatal intensive care unit

Discharge to
- O Home → Complete "Date of final discharge to home"
- O Other ward → Transfer date □□-□□-□□ (dd-mm-yy)
- O Other hospital

Admission #3

Type of admission
- O Ward
- O Medium care
- O Neonatal intensive care unit

Discharge to
- O Home → Complete "Date of final discharge to home"
- O Other ward → Transfer date □□-□□-□□ (dd-mm-yy)
- O Other hospital

Neonatal death before discharge home? O No O Yes

date □□-□□-□□ (dd-mm-yy)
time

Date of final discharge to home □□-□□-□□ (dd-mm-yy)

12. Neonatal diagnosis

Diagnosis as reported in the neonatal discharge letter of the pediatrician, all reported until 10 weeks after expected due date.

Chronic lung disease	O No	O Yes, no therapy<br>O Yes, only intubation/CPAP<br>O Yes, intubation/CPAP and surfactant
Necrotizing Enterocolitis (NEC)*[2]	O No	O Stage 1 O Stage 2 O Stage 3
Intracerebral haemorrhage (IVH)*[3]	O No	O Grade 1 O Grade 2 O Grade 3<br>O Grade 4 O Grade unknown
Periventricular leukomalacia (PVL)*[4]	O No	O Grade 1 O Grade 2 O Grade 3<br>O Grade 4 O Grade unknown
Retinopathy of Prematurity (ROP)	O No	O Yes, no therapy<br>O Yes, conservative treatment (e.g. oxygen or medical therapy)<br>O Yes, requiring surgery
Patent ductus arteriosus (PDA)	O No	O Yes, no therapy<br>O Yes, conservative treatment (e.g. medical therapy)<br>O Yes, requiring surgery
Treated seizures	O No	O Yes
Early neonatal sepsis (<72 hours)*[5]	O No	O Suspected<br>O Proven with positive culture
Late neonatal sepsis (>72 hours)*[6]	O No	O Suspected<br>O Proven with positive culture
Neonatal meningitis	O No	O Suspected<br>O Proven with positive culture
Other		Specify………………..

* Defined as:

1. infants with oxygen dependency at either 28 days of life or 36 weeks' gestation
2. Necrotizing Enterocolitis (NEC) > stage 1: the presence of the characteristic clinical features of abdominal distention, with or without rectal bleeding, and abdominal radiographic finding associated with pneumatosis intestinalis (this last finding is an abnormal gas pattern with dilated loops consistent with ileus). Staging according to Bell stages
3. intracerebral haemorrhage grade III or IV: IVH with ventricular dilation or parenchymal extension, confirmed by MRI, sonogram or CT scan
4. Periventricular lucency in the white matter.
5. If prior to or at 72 hours of life the infant had an infection marked by positive blood, CSF, or urine (catheterized or suprapubic) cultures with or without suspicious clinical findings of infection on physical examination.
6. If after 72 hours of life the infant had an infection marked by positive blood, CSF, or urine (catheterized or suprapubic) cultures with or without suspicious clinical findings of infection on physical examination.
   OR
   If there is clinical evidence of cardiovascular collapse or an unequivocal X-ray confirming infection and often cardiovascular decomposition

13. Serious Adverse Event

In this pregnancy has there been a Serious Adverse Event (SAE)?

O no O yes

|

☐ Perinatal death
☐ Maternal death
☐ Maternal hospitalization or prolongation of existing inpatients' hospitalization (not related to delivery or threatened preterm birth)

In this pregnancy has there been an Adverse Event (AE)?

O no O yes

|

☐ Preterm labor (before AD $34^{0/7}$)
☐ Severe neonatal morbidity (NICU admission)
☐ Necrotic cervix due to pessary

END OF Case Report Form

THANK YOU FOR FILLING OUT THIS FORM

15. End of study

Please specify if the patient completed the entire course of the study as specified in the protocol or discontinued the study:

O Completed

If continued, did patient withdrew consent after completing study?

O no O yes

If yes, date consent withdrawn: |__|__| |__|__|__| |__|__|__|__|
d d m m m y y y y

O Discontinued

If discontinued, please specify **the most appropriate** reason for early termination:

O Adverse event[2], please specify: …………………………………………………………………

O Patient lost to follow up
Date of last contact with patient in this study: |__|__| |__|__|__| |__|__|__|__|
d d m m m y y y y

O Patient withdrew consent
Date consent withdrawn: |__|__| |__|__|__| |__|__|__|__|
d d m m m y y y y
Reason: ………………………………………………………………………..

O Investigator's and/or physician's decision
Date of decision: |__|__| |__|__|__| |__|__|__|__|
d d m m m y y y y
Reason: ………………………………………………………………………..

O Other reason, please specify:

……………………………………………………………………………..……………………..

……………………………………………………………………………..……………………..
